# Maternal dissatisfaction with their children's body size in private schools in the Federal District, Brazil

**DOI:** 10.1371/journal.pone.0204848

**Published:** 2018-10-09

**Authors:** Jéssica Pedroso, Natacha Toral, Muriel Bauermann Gubert

**Affiliations:** Postgraduate Program in Human Nutrition, University of Brasília, Brasília, Federal District, Brazil; Indiana University Bloomington, UNITED STATES

## Abstract

We investigated the prevalence of maternal dissatisfaction with their child's body size and its associated factors among mothers of first- to third-grade elementary school students in private schools in the Federal District, Brazil. This is a cross-sectional study with 548 mother-schoolchildren pairs. We measured children’s weight and height, and collected their mother’s sociodemographic data and Body Mass Index using an online questionnaire. We also verified maternal body dissatisfaction and maternal dissatisfaction with their child's body size using Shape Scales. Most mothers (50.5%) were dissatisfied with their child's body size. Mothers of boys (Adjusted OR = 2.85) were more likely to want a larger silhouette for their child, while mothers of girls (Adjusted OR = 3.18), overweight (Adjusted OR = 24.83) and obese (Adjusted OR = 189.86) children were more likely to want a thinner silhouette for their child. A positive correlation was observed between maternal dissatisfaction with their own body and maternal dissatisfaction with their children's body size (r_s_ = 0.178). There was a high prevalence of maternal dissatisfaction with their child's body size, particularly among mothers of overweight and obese children. Additional studies should be conducted to better understand the influence of this dissatisfaction on maternal practices and attitudes related to their child’s body, food consumption, and lifestyle.

## Introduction

Family is known to hold meaningful influence over their children’s eating habits, and mothers play an important role on educating and selecting foods for their offspring [1–6]. Therefore, the way mothers perceive their child's body and the presence of maternal dissatisfaction with their child's body may affect their attitudes and practices related to the child's dietary intake [1,4,7–9].

Dissatisfaction occurs when parents believe that the child is above or below an idealized weight [10]. Evidence shows that maternal dissatisfaction is associated with the child's sex, age, and nutritional status [11–13]. Additionally, maternal characteristics and the way the mother perceives her own body may also influence the way she perceives her child's body [4,9,13,14].

Studies from Europe and Latin America show that mothers usually tend to idealize a larger silhouette for their children, as they consider it as a sign of health [4,9,15–17]. In Brazil, a study that investigated mother-toddler pairs verified that 43% of the mothers desired their children to be larger, including mothers of overweight children [17]. This misperception is an issue, especially concerning overweight children, because it can turn parents away from seeking a healthy nutritional status for their child [10].

This dissatisfaction can also generate inappropriate feeding practices, leading mothers to pressure their children to eat or use restrictive feeding behaviors. Both can negatively influence the child’s current and future food consumption [17,18]. Also, the way in which parents express dissatisfaction with their children's bodies may also affect the latter’s relationship with their own body and with food [19].

Therefore, the study of maternal dissatisfaction and its potential impact on practices and attitudes related to their children's dietary intake is relevant for both the creation of public policies that seek to prevent weight problems in children and for the guidance of health professionals [17]. Despite the importance of this topic for public health, to our knowledge no previous study has evaluated maternal dissatisfaction with schoolchildren’s body size in Brazil. Thus, the present study aims to break new ground and investigate the prevalence of maternal dissatisfaction with their children's body size and its associated factors in the Federal District, Brazil.

## Material and methods

A cross-sectional study was conducted with pairs of children and their mothers. Our sample of 548 children was representative of first- to third-grade elementary students attending private schools in the Federal District in 2013 [20] assuming a 95% confidence interval (95% CI) and a maximum error of 5%.

The study included children who lived with their mothers and were formally enrolled in selected private schools. In Brazil, families whose children attend private schools usually have higher income and education levels [21,22], which meant they were more likely to have Internet access and thus be able to respond to our online questionnaire.

We excluded mother-child pairs when: (i) the mother was pregnant; (ii) the child suffered from a disease or disability that could interfere in anthropometric measures or in the study’s outcome; (iii) the mother did not fill out the questionnaire in its entirety; or (iv) the child did not have his or her weight and height measured.

The present paper is part of a larger study that also evaluated maternal perception of children's nutritional status, as well as maternal attitudes, beliefs and practices related to children feeding. The first data on maternal perception of children's nutritional status has already been published [1]. The current analysis adds new understanding on the factors associated with maternal dissatisfaction with their schoolchildren’s body size.

### Procedures

A random list was generated to establish the order in which schools would be invited to participate in the study. At this point, all private schools located in the Federal District were eligible. The Federal District is where Brasilia, the capital of Brazil, is situated, and it is divided into thirty-one administrative regions. This study was conducted in nineteen schools spread across eleven of these regions, which were invited systematically according to the random list until an adequate sample size was met.

After the formal acceptance of participation of each school, we sent an invitation letter to eligible mothers presenting the study. The letter also contained a link that they should use to access the online questionnaire (available on the Survey Monkey platform) and their child’s code number (which allowed us to link the mother’s questionnaire to the child’s anthropometric data). The number of students interviewed in each school varied according to the number of enrolled students (the sample in each school ranged from 1 to 154 students). Invitation letters were also sent by email whenever possible. Mothers accepted participation in the study and authorized the involvement of their child by agreeing to a consent form available at the beginning of the questionnaire. This study was approved by the Research Ethics Committee of the School of Health Sciences at University of Brasilia under no. 39116314.3/0000.0030.

In addition to their mothers’ consent, children were also required to accept participation in the study by signing a written informed consent form. They then had their weight and height measured at school by a trained researcher at a previously scheduled date. We used a calibrated portable scale and a stadiometer for these measurements. The children’s BMI was calculated and their nutritional status classified according to BMI-for-age z-scores, using the AnthroPlus software, with a cutoff point of z-score > +1 and ≤ +2 for overweight and z-score >+ 2 for obesity [23].

The questionnaire's adequacy was assessed in a pilot test performed in a few private schools not selected for the study. The questionnaire evaluated sociodemographic data, maternal self-reported weight and height, maternal dissatisfaction with their own body size, and maternal dissatisfaction with their child's body size. The following sociodemographic data was collected: (i) child's age and sex; (ii) maternal age, education level, and marital status; and (iii) family income. Maternal BMI status was calculated using mothers’ self-reported weight and height, a procedure that has been validated and used in annual population inquiries conducted in Brazil, being a reliable data and showing a good agreement when compared with measured weight and height [24–26]. We used the World Health Organization’s cutoff points to classify mothers’ BMIs: ≥ 25 kg/m^2^ and < 30 kg/m^2^ for overweight and ≥ 30 kg/m^2^ for obesity [27].

Maternal body dissatisfaction was evaluated using a silhouette scale for female adults [28] (S1 Fig), which presents fifteen silhouettes ranging from very thin (‘Silhouette 1’, with a mean BMI of 12.5 kg/m^2^) to severely obese (‘Silhouette 15’, with a mean BMI of 47.5 kg/m^2^). First, mothers were asked to identify among these silhouettes the one that best represented their current body, which we named perceived maternal silhouette (PMS). Then they were asked to choose the one that best represented the body they wanted to have, which we named desired maternal silhouette (DMS). The difference in number of silhouettes between the PMS and the DMS was used to determine the level of maternal body dissatisfaction. If the difference was equal to zero, mothers were classified as satisfied with their own size. A positive difference indicated that they desired a thinner silhouette (a ‘maternal thinner silhouette’, or MTS), whereas a negative difference indicated that mother desired a larger silhouette (a ‘maternal larger silhouette’, or MLS).

Maternal dissatisfaction with their child's body size was assessed using a silhouette scale for children [28] (S1 Fig), which presents twenty-two silhouettes (eleven male and eleven female) ranging from very thin (‘Silhouette 1’, with a mean BMI of 12.0 kg/m^2^) to severely obese (‘Silhouette 11’, with a mean BMI of 29.0 kg/m^2^). First, mothers were asked to identify, among the 11 silhouettes, the one that best represented the current body of their child, which we named perceived child's silhouette (PCS). Then they were asked to choose the one that best represented the body they wanted their child to have, which we named desired child's silhouette (DCS). The difference in numbers of silhouettes between the PCS and the DCS was used to determine the level of maternal dissatisfaction with child's body size. If the difference was equal to zero, mothers were classified as satisfied with her child's body size. A positive difference between the two indicated that they wanted their child to be thinner (a ‘child thinner silhouette’, or CTS), whereas a negative difference indicated that they wanted their child to be larger (a ‘child larger silhouette’, or CLS).

These silhouette scales were developed and validated for the Brazilian population [28,29]. The measurement of dissatisfaction using the difference between desired and perceived body size was previously used in other studies [9,30].

### Statistical analyses

Pairs with underweight children or mothers were excluded because of their low prevalence in the sample (n = 10). Descriptive analyses of the sample were performed (mean, standard deviation, and frequency distribution).

The variables were categorized in the follow categories: child's age (5–6, 7 and 8–9 years old); child's and mother’s nutritional status (classified as normal weight, overweight or obese); maternal age (≤ 35 or ≥ 36 years old); marital status was recodify in single-parent household (single, divorced, separated or widowed mother) or two-parents households; maternal educational level (complete higher education or less or postgraduate education or above); monthly family income, considering a minimum wage of US$ 210 (< 9 minimum wages, US$ 1890; 9–15 minimum wages, US$ 1890–3159; or >15 minimum wages, US$ 3159); and maternal body dissatisfaction (presence/absence of desire for MLS and presence/absence of desire for MTS).

The chi-square test was used to evaluate the association between sociodemographic, maternal, and children's variables, with the outcome variables being: (i) dissatisfaction with their child's body size (wanted CLS and CTS); (ii) wanted CLS; and (iii) wanted CTS.

A multivariate analysis with logistic regression was performed to evaluate maternal dissatisfaction with their child's body size, considering unadjusted and adjusted odds ratios (95% CI). This multivariate analysis included only the variables with p ≤ 0.20 at the bivariate analysis, and therefore, different independent variables were chosen for each model based on the previous analysis.

Child's sex, child's age, child's nutritional status, maternal nutritional status, maternal educational level, family income, and maternal body dissatisfaction were used as control variables in the model for maternal dissatisfaction with child's body size. Child's sex, child's age, child's nutritional status, maternal age, and maternal body dissatisfaction were used as control variables in the model for desire for CLS. On the other hand, child's sex, child's age, child's nutritional status, maternal nutritional status, maternal educational level, family income, and maternal body dissatisfaction were used as control variables in the model for desire for CTS.

Spearman correlation was used to investigate the existence of correlation between maternal body dissatisfaction and maternal dissatisfaction with their child's body size. Analyses were conducted using the Statistical Package for the Social Sciences software version 20.0, considering a level of significance of 5% and a confidence interval of 95%.

## Results

Children's mean age was 7.1 ± 0.8 years. Of these, 21.3% were overweight and 12.8% were obese (Table 1). Mothers' mean age was 37.6 ± 5.1 years, and the prevalence of overweight and obesity was 28.7% and 11.3% respectively (Table 1). Additionally, 65.5% were thirty-six years old or older, and 42.9% of mothers in the sample had a family income above fifteen minimum wages (Table 1).

**Table 1:** Descriptive analysis of mothers and their respective children, 548 first- to- third-grade elementary school students in private schools. Brasília (DF). 2015.

Study variables	n	%
**Child's sex**		
Male	279	50.9
Female	269	49.1
**Child's nutritional status**		
Normal weight	361	65.9
Overweight	117	21.3
Obese	70	12.8
**Maternal nutritional status**		
Normal weight	329	60.0
Overweight	157	28.7
Obese	62	11.3
**Maternal age**		
≤ 35 years	189	34.5
≥ 36 years	359	65.5
**Marital status**		
Single/divorced/separated/widowed	70	12.8
Married/living with a partner	478	87.2
**Maternal education level**		
Higher education and below	274	50.0
Postgraduate education and above	274	50.0
**Family income**		
Below nine minimum wages	173	31.6
Nine to fifteen minimum wages	140	25.5
Above fifteen minimum wages	235	42.9

### Maternal dissatisfaction with perceived child's silhouette

Analysis of the sample revealed that 50.5% of mothers were dissatisfied with their child's body size (26.6% wanted CLS and 23.9% wanted CTS). Most mothers of normal weight children (57.1%) were satisfied with their child's body size, but 38.5% of them wanted CLS. Regarding overweight children, 46.2% of mothers claimed to be satisfied with their child's body size, whereas 48.7% of them expressed the desire for CTS and 5.1% for CLS. The vast majority of mothers (82.9%) of obese children idealized a thinner silhouette for their child (Table 2).

**Table 2:** Presence or absence of maternal satisfaction with their child's body size, maternal desire for a child thinner silhouette and maternal desire for child larger silhouette in a sample of 548 first- to third-grade elementary school students in private schools. Brasília (DF). 2015.

	Satisfied	Want Child Larger Silhouette (CLS)	Want Child Thinner Silhouette (CTS)	Total
	n (%)	n (%)	n (%)	n (%)
**Overall sample**	271 (49.5)	146 (26.6)	131 (23.9)	548 (100.0)
**Child's nutritional status**				
Normal weight	206 (57.1)	139 (38.5)	16 (4.4)	361 (100.0)
Overweight	54 (46.2)	6 (5.1)	57 (48.7)	117 (100.0)
Obese	11 (15.7)	1 (1.4)	58 (82.9)	70 (100.0)

### Maternal body dissatisfaction

Examining maternal body dissatisfaction (the difference between PMS and DMS), it was found that only 12.4% of mothers were satisfied with their body size. Most mothers (79.7%) wanted to lose weight, even though only 40.0% of the sample mothers were overweight or obese (data not shown in tables).

### Bivariate analysis of maternal dissatisfaction with perceived child's silhouette

It was observed that mothers were more likely to be dissatisfied with the body size of male children (55.6%). Furthermore, mothers of boys were more likely to want them to gain weight (CLS) (34.4%) than mothers of girls, as were mothers of younger children (32.4%) when compared to older offspring (Table 3).

**Table 3:** Bivariate association of sociodemographic and maternal variables with the presence or absence of maternal dissatisfaction, desire for a child larger silhouette, and desire for a child thinner silhouette. Brasília (DF). 2015.

Variables	Dissatisfied	Satisfied	*p*	Want Child Larger Silhouette	Do not want Child Larger Silhouette	*p*	Want Child Thinner Silhouette	Do not want Child Thinner Silhouette	*p*
	n(%)	n(%)		n(%)	n(%)		n(%)	n(%)	
**Child's sex**			0.017			<0.001			0.123
Male	155 (55.6)	124 (44.4)		96 (34.4)	183 (65.6)		59 (21.1)	220 (78.9)	
Female	122 (45.4)	147 (54.6)		50 (18.6)	219 (81.4)		72 (26.8)	197 (73.2)	
**Child's age**			0.092			0.047			0.039
5–6 years	71 (49.0)	74 (51.0)		47 (32.4)	98 (67.6)		24 (16.6)	121 (83.4)	
7 years	114 (56.4)	88 (43.6)		57 (28.2)	145 (71.8)		57 (28.2)	145 (71.8)	
8–9 years	92 (45.8)	109 (54.2)		42 (20.9)	159 (79.1)		50 (24.9)	151 (75.1)	
**Child's nutritional status**			<0.001			<0.001			<0.001
Normal weight	155 (42.9)	206 (57.1)		139 (38.5)	222 (61.5)		16 (4.4)	345 (95.6)	
Overweight	63 (53.8)	54 (46.2)		6 (5.1)	111 (94.9)		57 (48.7)	60 (51.3)	
Obese	59 (84.3)	11 (15.7)		1 (1.4)	69 (98.6)		58 (82.9)	12 (17.1)	
**Maternal nutritional status**			<0.001			0.948			<0.001
Normal weight	143 (43.5)	186 (56.5)		86 (26.1)	243 (73.9)		57 (17.3)	272 (82.7)	
Overweight	96 (61.1)	61 (38.9)		43 (27.4)	114 (72.6)		53 (33.8)	104 (66.2)	
Obese	38 (61.3)	24 (38.7)		17 (27.4)	45 (72.6)		21 (33.9)	41 (66.1)	
**Maternal age**			0.533			0.079			0.275
≤ 35 years	99 (52.4)	90 (47.6)		59 (31.2)	130 (68.8)		40 (21.2)	149 (78.8)	
≥ 36 years	178 (49.6)	181 (50.4)		87 (24.2)	272 (75.8)		91 (25.3)	268 (74.7)	
**Marital status**			0.723			0.496			0.263
**Single/divorced/separated/widowed**	34 (48.6)	36 (51.4)		21 (30.0)	49 (70.0)		13 (18.6)	57 (81.4)	
Married/living with a partner	243 (50.8)	235 (49.2)		125 (26.2)	353 (73.8)		118 (24.7)	360 (75.3)	
**Maternal education level**			0.013			0.699			0.001
Complete higher education and below	153 (55.8)	121 (44.2)		71 (25.9)	203 (74.1)		82 (29.9)	192 (70.1)	
Postgraduate education and above	124 (45.3)	150 (54.7)		75 (27.4)	199 (72.6)		49 (17.9)	225 (82.1)	
**Family income**			0.065			0.548			0.173
Below nine minimum wages	99 (57.2)	74 (42.8)		49 (28.3)	124 (71.7)		50 (28.9)	123 (71.1)	
Nine to fifteen minimum wages	71 (50.7)	69 (49.3)		40 (28.6)	100 (71.4)		31 (22.1)	109 (77.9)	
Above fifteen minimum wages	107 (45.5)	128 (54.5)		57 (24.3)	178 (75.7)		50 (21.3)	185 (78.7)	
**Maternal body dissatisfaction**			0.175			<0.001			0.019
Want a larger silhouette (MLS)	26 (60.5)	17 (39.5)		22 (51.2)	21 (48.8)		4 (9.3)	39 (90.7)	
Do not want a larger silhouette	251 (49.7)	254 (50.3)		124 (24.6)	381 (75.4)		127 (25.1)	378 (74.9)	
**Maternal body dissatisfaction**			0.194			0.023			<0.001
Want a thinner silhouette (MTS)	227 (51.9)	210 (48.1)		107 (24.5)	330 (75.5)		120 (27.5)	317 (72.5)	
Do not want a thinner silhouette	50 (45.0)	61 (55.0)		39 (35.1)	72 (64.9)		11 (9.9)	100 (90.1)	

Mothers of overweight (53.8%) and obese children (84,3%) were more dissatisfied with their child's body size and were more likely to desire CTS (48.7% and 82.9%, respectively), whereas mothers of normal weight children were more likely to desire the opposite (38.5%).

Overweight (61.1%) and obese mothers (61.3%) were more dissatisfied with their child's body size compared to normal weight mothers, and were more likely to desire CTS (33.8% and 33.9%, respectively) (Table 3). Less educated mothers were more dissatisfied with their child's body size (55.8%) and were more likely to look for CTS (29.9%). It was observed that mothers who desired to gain weight were more likely to want their child to gain weight (51.2%), whereas mother who desired to lose weight were more likely to want their child to lose weight (27.5%) (Table 3). Moreover, a significant positive correlation was observed between maternal dissatisfaction with their child's body size and maternal body dissatisfaction (r_s_ = 0.178, 95% CI [0.092–0.259], p < 0.001).

### Multivariate analyses of maternal dissatisfaction with perceived child's silhouette

#### Maternal dissatisfaction

The variables that remained significantly associated with dissatisfaction with child's body size after adjusting the model were child's age, child's nutritional status, maternal nutritional status, and desire for MLS (Table 4).

**Table 4:** Unadjusted and adjusted odds ratio for the presence of body weight dissatisfaction, maternal desire for a child larger silhouette, and maternal desire for a child thinner silhouette, according to associated factors. Brasília (DF). 2015.

	Presence of dissatisfaction		Maternal desire for Child Larger Silhouette (CLS)		Maternal desire for Child Thinner Silhouette (CTS)	
**Associated factors**	OR (95%CI)	Adjusted OR (95%CI)*	OR (95%CI)	Adjusted OR (95%CI)^†^	OR (95%CI)	Adjusted OR (95%CI)^‡^
**Child's sex**						
**Male**	1.51	1.33	2.30	2.85	1	1
	(1.08–2.11)	(0.93–1.91)	(1.55–3.41)	(1.83–4.42)		
**Female**	1	1	1	1	1.36	3.18
					(0.92–2.02)	(1.70–5.95)
**Child's age**						
5–6 years	1.14	1.22	1.82	1.72	1	1
	(0.74–1.74)	(0.77–1.92)	(1.12–2.95)	(0.99–2.98)		
7 years	1.53	1.65	1.49	1.73	1.98	2.01
	(1.04–2.27)	(1.08–2.51)	(0.94–2.35)	(1.03–2.91)	(1.16–3.38)	(0.92–4.36)
8–9 years	1	1	1	1	1.67	1.65
					(0.97–2.87)	(0.76–3.59)
**Child's nutritional status**						
Normal weight	1	1	1	1	1	1
Overweight	1.55	1.35	0.09	0.07	20.48	24.83
	(1.02–2.36)	(0.87–2.09)	(0.04–0.20)	(0.03–0.16)	(11.03–38.02)	(12.50–49.31)
Obese	7.13	6.10	0.02	0.02	104.22	189.86
	(3.62–14.02)	(3.05–12.21)	(0.00–0.17)	(0.00–0.13)	(46.90–231.59)	(72.23–498.87)
**Maternal nutritional status**^§^						
Normal weight	1	1			1	1
Overweight	2.05	1.98			2.43	2.08
	(1.39–3.02)	(1.29–3.05)			(1.57–3.76)	(1.07–4.05)
Obese	2.06	1.58			2.44	0.81
	(1.18–3.59)	(0.86–2.91)			(1.34–4.45)	(0.33–2.04)
**Maternal age**^§^						
≤ 35 years			1.42	1.27		
			(0.96–2.10)	(0.80–2.01)		
≥ 36 years			1	1		
**Maternal education level**^§^						
Complete higher education and below	1.53	1.37			1.96	3.57
	(1.09–2.14)	(0.93–2.04)			(1.31–2.93)	(1.81–7.00)
Postgraduate education and above	1	1			1	1
**Family income**^§^						
Below nine minimum wages	1.60	1.19			1.50	0.76
	(1.08–2.38)	(0.75–1.89)			(0.96–2.37)	(0.37–1.59)
Nine to fifteen minimum wages	1.23	1.06			1.05	0.87
	(0.81–1.87)	(0.67–1.67)			(0.63–1.75)	(0.41–1.84)
Above fifteen minimum wages	1	1			1	1
**Maternal body dissatisfaction**						
Desired a larger silhouette (MLS)	1.55	2.51	3.22	4.40	0.30	0.63
	(0.82–2.92)	(1.10–5.73)	(1.71–6.05)	(1.71–11.29)	(0.11–0.87)	(0.11–3.42)
Did not desire a larger silhouette	1	1	1	1	1	1
**Maternal body dissatisfaction**						
Desired a thinner silhouette (MTS)	1.32	1.35	0.60	1.44	3.44	2.16
	(0.87–2.00)	(0.76–2.42)	(0.38–0.94)	(0.76–2.73)	(1.78–6.64)	(0.65–7.13)
Did not desire a thinner silhouette	1	1	1	1	1	1

OR, Odds ratio. CI, Confidence interval. Adjusted OR*: odds ratio adjusted by logistic regression for child's sex, child's age, child's nutritional status, maternal nutritional status, maternal education level, family income, and maternal body dissatisfaction.

Adjusted OR^†^: odds ratio adjusted by logistic regression for child's sex, child's age, child's nutritional status, maternal age, and maternal body dissatisfaction.

Adjusted OR^‡^: odds ratio adjusted by logistic regression for child's sex, child's age, child's nutritional status, maternal nutritional status, maternal education level, family income, and maternal body dissatisfaction.

^§:^ Variables not tested in all models because they had p > 0.20 in bivariate analyses.

Mothers of 7-year-old children were 65% more likely to be dissatisfied with their child's body size than mothers of 8- and 9-year-old children. It was also observed that mothers of obese children were more likely to be dissatisfied with their child's body size (adjusted OR = 6.10) and that overweight mothers were almost twice more likely to be dissatisfied with their child's body size (adjusted OR = 1.98). In turn, mothers who wanted to gain weight were 2.51-fold more likely to be dissatisfied with their child's body size.

#### Desire for child's weight gain

Mothers of boys were 2.85-fold more likely to want CLS. Mothers of overweight (adjusted OR = 0.07) and obese children (adjusted OR = 0.02) were less likely to desire CLS. Mothers who wanted to gain weight were 4.40-fold more likely to desire CLS. Mothers of 7-year-old children were 73% more likely to desire CLS.

#### Desire for child's weight loss

Child's sex, child's nutritional status, maternal nutritional status and maternal education level were the variables that remained associated with desire for CTS after adjustment. Mothers of girls were 3.18 times more likely to want CTS compared to mothers of boys. Mothers of overweight (adjusted OR = 24.83) and obese children (adjusted OR = 189.86) were more likely to want CTS. Moreover, overweight mothers were twice more likely to want CTS compared to normal weight mothers. Additionally, less educated mothers were also more likely to desire CTS (adjusted OR = 3.57) compared to highly educated ones.

## Discussion

An analysis of maternal dissatisfaction with their child's body size revealed that half of the mothers were indeed unsatisfied. This was expected, since we found a high prevalence of misperception of child’s nutritional status in previous study with the same mothers, when only 30.0% of them chose the appropriate silhouette that represented their children’s nutritional status [1]. Aparício et al. [4], when studying preschool children, observed a higher percentage of maternal satisfaction with their child's body compared with that of the present study (67.2%). Conversely, Duchin et al. found that only 39.0% of mothers were satisfied with the body of their child, whereas 47.0% wanted their child to have a larger silhouette [9].

It is worrisome that more than a third of mothers of normal weight children wanted a larger silhouette for their children in our study, given that satisfaction or dissatisfaction with the child's body may directly influence maternal practices and attitudes towards child feeding [4,9]. The desire for weight gain in normal weight children may lead mothers to pressure the child to eat a greater amount of food, which may conceal innate hunger and satiety cues and cause the child to eat in response to external stimuli, a behavior that may result in future overweight and obesity [7,18,31,32]. Therefore, it is important for further studies to expand upon this phenomenon.

As was expected, a greater percentage of mothers of overweight and obese children wanted their child to lose weight. Killion et al. observed that half the mothers of overweight children looked for CTS [30]. Guendelman et al. assessed Mexican-origin mothers living in California and found that, among the mothers who perceived their child as overweight, 82.3% were dissatisfied with their child’s weight [33]. It has been suggested that accurate perception of their children’s inadequate nutritional status may lead mothers to improve their child's eating habits and lifestyle. However, further studies are needed to confirm this correlation, as the present study was unable to verify it.

Most of the mothers in the study reported a desire for maternal thinner silhouette. Similar study [9] echoed these results in observing a high percentage of mothers dissatisfied with their own nutritional status (of those, over half wanted to have a smaller silhouette). The internalization of thinness ideals and the sociocultural pressure for women to be thin may be related to this high percentage of women's dissatisfaction with their bodies, and may lead to consequences such as low self-esteem, depression, and eating disorders [34,35]. This social obsession over slim silhouettes may affect the way women perceive their children's bodies and the way they interact with their offspring, which can lead to the adoption of strategies meant to control their child's body weight [3,4].

We found that mothers of girls were more likely to desire for child’s weight loss, whereas mothers of boys were more likely to desire for child’s weight gain. These findings are similar to those found in a previous study [4]. Maternal dissatisfaction with girls' bodies may lead mothers to press their daughters to engage in practices meant to reduce their body weight [36]. According to Schreiber et al., normal weight girls were more pressured by their mothers to seek weight loss than normal weight boys [37]. Mothers may want their sons to gain weight because they believe that larger silhouettes represent stronger, more robust bodies [3,38]. This seems to be the current beauty standard for males, an issue that may be delved into in further studies. It is important to emphasize that the children included in this study are prepubertal, and that pressuring them to reach robust bodies at such a young age is an unhealthy practice. This may lead them to become dissatisfied with their bodies as they grow up or to resort to supplements and steroids at an older age [39].

Mothers of overweight and obese children were the ones who most desired child thinner silhouette. This preference for thinner silhouettes by mothers of overweight children was also observed in previous studies [13,15]. Warschburger and Kröller [13] found an association between the desire for child’s weight loss and parental perception of the need for activities to prevent overweight in the child's routine. However, since the present study was not able to explore this topic, further studies should be conducted to investigate whether this perception may somehow encourage treatments or the adoption of a healthier lifestyle.

We found that less educated mothers were more likely to want their children to lose weight. These findings come as a contrast to Aparício et al. [4], who verified that less educated mothers tended to want their child to gain weight. This discrepancy may have been due to the high education level of all the sample mothers participating in our study, while Aparício et al. [4] had a more diverse sample.

It was also observed that overweight mothers were more likely to be dissatisfied with their child's nutritional status and to desire a CTS. Previous study found that overweight mothers preferred thinner silhouettes for their children, and suggested that overweight and obese mothers may be more aware of changes in their children's bodies and grow more concerned about weight gain, due to their personal experience [13]. Conversely, mothers who wanted to gain weight were more likely to want their children to gain weight, a finding previously observed in a similar study [9]. Mothers who worried about their own weight were more likely to encourage their children to control their body weight [37], a practice that may be detrimental to the child's health and is associated with future eating disorders.

Although the present study evaluated only maternal dissatisfaction with their children’s body size, it is relevant to highlight the importance of paternal dissatisfaction with their own body size and with their children’s body size as well, since paternal factors can influence feeding practices and child body satisfaction, eating and body size attitude [38, 40].

This study provides novel data about maternal dissatisfaction with Brazilian schoolchildren’s body size using a scale of silhouettes developed specifically for the Brazilian population. However, it was limited by the difficulty in generalizing its results to broader populations, due to the high maternal education level and income of our study population. Besides that, although the adopted definition of dissatisfaction (the existence of a difference between the perceived and the desired silhouette equal to zero) is widely used in previous studies [4,9,10,12,13,15,16,30], it may have overestimated the number of mothers dissatisfied with their own body and with their children’s bodies, since the degree of dissatisfaction was not evaluated. Also, the silhouette scale only evaluated maternal dissatisfaction towards child size, not taking into account possible dissatisfaction with height or muscularity.

## Conclusion

The present study found that most mothers were dissatisfied with their child's body size. Additionally, mothers of boys and of were more likely to want CLS, whereas mothers of girls and of overweight and obese children were more likely to want CTS. A positive correlation was observed between maternal body dissatisfaction and dissatisfaction with child's body size. This study suggests that mothers tend to transpose their body dissatisfaction to their child's body, as well as their desire to lose or gain weight. Thus, the study findings can help guide health professionals and policy makers to develop and improve interventions and public policies that seek to prevent weight problems in children.

Within this context, the discussion about current health and beauty standards and their impact on the perception of child's nutritional status gains relevance. Furthermore, maternal involvement in strategies to prevent and treat childhood overweight is relevant, and maternal concerns and dissatisfactions with her body and with her child's body should be considered. Therefore, it is necessary that health professionals make an integral approach because the mother’s dissatisfaction with child’s body size can have a potential impact on maternal attitudes and practices related to children's food intake and on the relationship of children with their own body size as they mature. Moreover, longitudinal studies should be conducted to evaluate the influence of maternal dissatisfaction with child's body size on maternal practices and attitudes related to their child’s body, food consumption and lifestyle.

## Supporting information

S1 Dataset Maternal dissatisfaction with child’s body dataset. (XLSX)

S1 Fig Silhouette scale for female adults and for children. (PDF)
