# Supplementary material for: Maternal dissatisfaction with their children's body size in private schools in the Federal District, Brazil
Source: PLoS One. 2018 Oct 9;13(10):e0204848. doi: 10.1371/journal.pone.0204848 (PMC6177138; doi:10.1371/journal.pone.0204848)
Supplement: S1 Fig — (PDF) [file pone.0204848.s002.pdf]

Silhouette scale for female adults

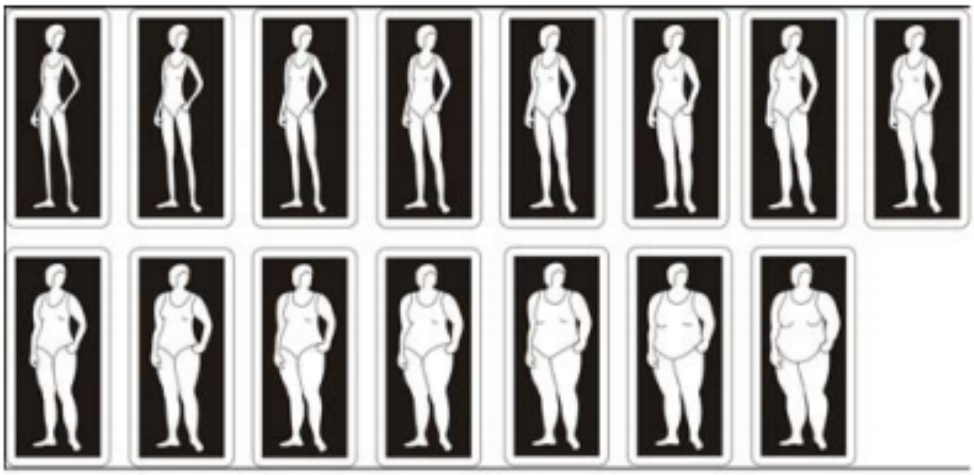

Silhouette scale for children

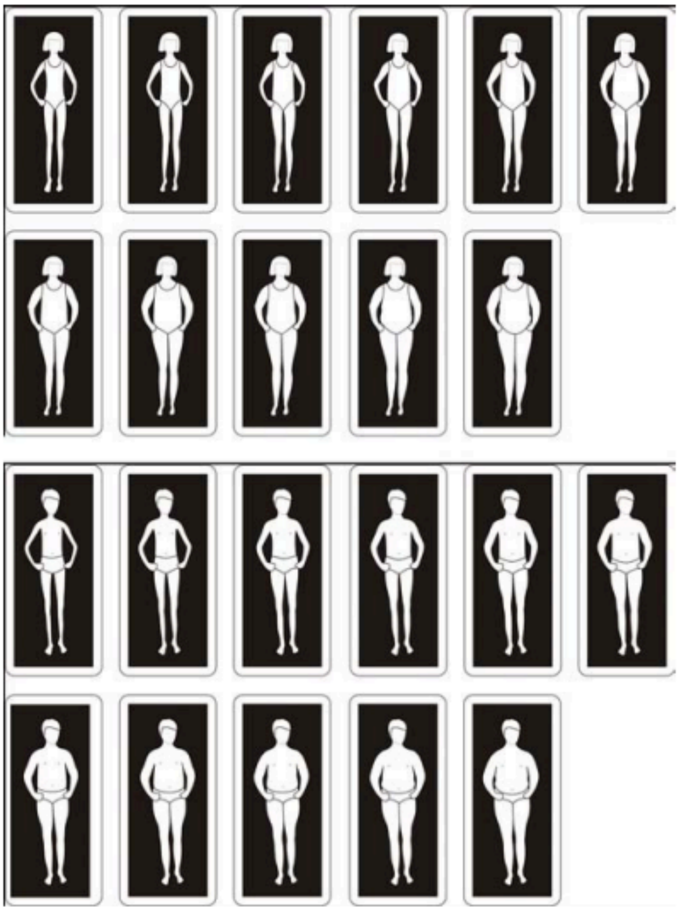

Reference

Kakeshita IS, Silva AIP, Zanatta DP, Almeida SS. Construção e fidedignidade teste-reteste de escalas de silhuetas brasileiras para adultos e crianças. Psicologia: Teoria e Pesquisa. 2009;25(2): 263-270.
